# Pterostilbene in Cancer Therapy

**DOI:** 10.3390/antiox10030492

**Published:** 2021-03-21

**Authors:** Elena Obrador, Rosario Salvador-Palmer, Ali Jihad-Jebbar, Rafael López-Blanch, Thanh H. Dellinger, Ryan W. Dellinger, José M. Estrela

**Affiliations:** 1 Department of Physiology, Faculty of Medicine and Odontology, University of Valencia, 15 Av. Blasco Ibañez, 46010 Valencia, Spain; elena.obrador@uv.es (E.O.); rosario.salvador@uv.es (R.S.-P.); aji.jebbar@gmail.com (A.J.-J.); loblanch@alumni.uv.es (R.L.-B.); 2 Department of Surgery, Division of Gynecologic Surgery, City of Hope, 1500 East Duarte Rd, Duarte, CA 91010, USA; tdellinger@coh.org; 3 Elysium Health Inc., 434 Broadway, New York, NY 10013, USA; ryan@elysiumhealth.com

**Keywords:** pterostilbene, polyphenols, stilbenes, cancer, oxidative stress, heat-shock proteins

## Abstract

Natural polyphenols are organic chemicals which contain phenol units in their structures and possess antitumor properties. However, a key problem is their short half-life and low bioavailability under in vivo conditions. Pterostilbene (3,5-dimethoxy-4′-hydroxystilbene; PT) is a phytoalexin originally isolated from the heartwood of red sandalwood. As recently reported by our group, PT was shown to be effective in the treatment of melanoma. Counterintuitively, PT is not effective (cytotoxic) against melanoma in vitro, and only under in vivo conditions does PT display its anticancer activity. This study elucidated that PT can be effective against melanoma through the inhibition of adrenocorticotropic hormone production in the brain of a mouse, which weakens the Nrf2-dependent antioxidant defenses of melanoma and also pancreatic cancers. This results in both the inhibition of tumor growth and sensitization of the tumor to oxidative stress. Moreover, PT can promote cancer cell death via a mechanism involving lysosomal membrane permeabilization. Different grades of susceptibility were observed among the different cancer cells depending on their lysosomal heat shock protein 70 content, a known stabilizer of lysosomal membranes. In addition, the safety of PT administered i.v. has been evaluated in mice. PT was found to be pharmacologically safe because it showed no organ-specific or systemic toxicity (including tissue histopathologic examination and regular hematology and clinical chemistry data) even when administered i.v. at a high dose (30 mg/kg per day × 23 days). Moreover, new pharmacological advances are being developed to increase its bioavailability and, thereby, its bioefficacy. Therefore, although applications of PT in cancer therapy are just beginning to be explored, it represents a potential (and effective) adjuvant/sensitizing therapy which may improve the results of various oncotherapies. The aim of this review is to present and discuss the results that in our opinion best support the usefulness of PT in cancer therapy, making special emphasis on the in vivo evidence.

## 1. Introduction

At present, combined therapies involving distinct molecular mechanisms are considered the most promising strategies for cancer treatment. Although such combinations have shown efficacy in preclinical models, the results in clinical trials have not been encouraging in many cases. This suggests that malignant cells (likely particular cell subsets), treated to block specific pathways, find ways to adapt using alternative survival mechanisms. Based on available experimental evidences, by increasing the efficacy of biotherapy, cytotoxic drugs, or ionizing radiation, and/or by directly promoting cancer cell death, pterostilbene (PT) may be a useful agent to treat different established cancers. This prompted us to update which evidence can support the potential use of PT in clinical oncotherapy. 

PT (3,5-dimethoxy-4′-hydroxystilbene) is a polyphenol (compounds derived from the shikimate/phenylpropanoid and/or the polyketide pathway, featuring more than one phenolic unit and without nitrogen-based functional groups) as well as a phytoalexin (antimicrobial substance synthesized de novo by plants). PT is also the natural analog of resveratrol (3,5,4′-trihydroxystilbene), but is a much stronger antifungal agent (>10 times) than resveratrol. Moreover, PT, with two methoxy groups and one hydroxyl group, has greater lipophilicity and a higher potential for cellular uptake than resveratrol, which has three hydroxyl groups [1].

PT, a secondary metabolite of plants originally isolated from the heartwood of red sandalwood (*PTocarpus santalinus*), is also found in other plants and berries, e.g., blueberries (approx. 10–15 mg/kg of fresh weight) [2,3]. A resveratrol O-methyltransferase, which catalyzes the synthesis of PT from resveratrol, was identified in grapevine leaves where it is induced by different types of stress [4]. Indeed, PT is involved (as are other natural polyphenols) in plant defense against different stressful conditions, i.e., UV radiation, aggression by pathogens, low soil fertility, high/low temperatures, severe drought, or grazing pressure [5,6].

Based on reported experimental evidence, PT has potential health benefits in inflammatory dermatoses, photoprotection, cancer prevention and therapy, insulin sensitivity, blood glycemia and lipid levels, cardiovascular diseases, aging, and memory and cognition [7,8].

Cancer cells exhibit high levels of oxidative stress, as compared to their normal counterparts [9]. A rise in intracellular reactive oxygen species (ROS) levels has two potentially important effects: damage to various cell components and triggering of the activation of specific signalling pathways. Both effects can influence numerous cellular processes linked to cancer progression. ROS have been shown to promote the proliferation of cancer cells, which highlights their cancer promoting potential. The exposure of several cancer cell lines to inflammation- or chemically induced ROS boosts their migratory and invasive behaviors, hence suggesting a role of these reactive species in favoring the invasive phenotype. Besides, it is well known that exposure to ROS above a certain threshold irreversibly leads to cell damage, and eventually to cell death [10,11,12]. Therefore, the net result of pro- and anti-cancer ROS effects may likely determine the rate and extent of in vivo tumor progression. Based on this reasoning, elevated rates of intrinsic reactive oxygen species (ROS) generation may confer a higher susceptibility of cancer cells to further oxidative stress induced by treatments. However, due to the innate characteristics of in vivo growing cancers, this strategy could facilitate the selection of cancer cell subclones highly resistant to therapies. In this scenario, increased ROS generation could contribute to the ability of cancers to mutate, injure local tissues, and promote tumor heterogeneity and metastases [13]. Paradoxically, by reducing the redox stress in cancer cells, antioxidant supplements (and pharmaceuticals) could decrease the effectiveness of radiotherapy and chemotherapy. However, as reported by our group, PT was shown to be effective in the treatment of malignant melanoma. Counterintuitively, PT was not found effective (cytotoxic) in vitro, only in vivo. PT can be effective against melanoma by decreasing adrenocorticotropin hormone (ACTH), which interferes in the Nrf2-dependent activity in the metastatic cells [14]. Nrf2 is a transcription factor that regulates the expression of antioxidant and defense proteins that protect against oxidative damage triggered by injury and inflammation [14]. In fact, in many cancer cells the Keap1-mediated Nrf2 downregulation is abolished or diminished, thus generating an addiction to Nrf2 and, thereby, favoring cancer cell defenses and progression [15].

## 2. Metabolism and Pharmacokinetics

Regarding potential applications of PT in cancer therapy, its administration (depending on the indication) can be carried out orally, i.v., or topically.

Orally administered polyphenols, including PT, undergo rapid conjugation in the intestinal tract of humans and rodents. Polyphenol aglycones, when absorbed by intestinal enterocytes, undergo extensive phase II metabolism via uridine 5’-diphospho-glucuronosyltransferase isoforms. Conjugates are absorbed with very little of the free chemical structure gaining access to the systemic blood circulation. Moreover, polyphenol aglycones present in the blood undergo further (and also rapid) metabolism in the liver to methylated, glucuronidated, and/or sulfated conjugates. These features, as well as the mechanisms of elimination in the urine and via the biliary tract, as well as recycling by the intestinal tract, have been reviewed in detail (e.g., Estrela 2013, Liu 2020) [7,8]. 

Available data strongly suggest that natural polyphenols and their derivatives are biologically more active than their metabolites. Therefore, orally administered polyphenols are unlikely to be systemically effective unless their biological effects are not inactivated by conjugation, and/or the free polyphenol can be released by the hydrolysis of conjugates and taken up by cancer cells in sufficient amounts to generate pharmacologically active concentrations. These limitations may be minimized in the case of primary tumors of the gastrointestinal tract where the orally administered polyphenol can directly reach the growing tumor.

Alternatively, PT can be administered i.v. When 20 mg/kg of PT was administered i.v. to mice, pharmacokinetics studies showed a peak of approximately 95 μM in plasma 5 min after administration, which then rapidly decreased to about 20 μM at 60 min and 2 μM at 240 min. Potential recycling did not further increase PT plasma levels (approx. 1 μM 480 min after i.v. administration) [16]. Plasma and total blood levels were not significantly different [16]. Tumor levels of PT, following i.v. administration, were even lower. For example, after the i.v. administration of PT at 30 mg/kg, the highest concentration range observed in the plasma of nude mice bearing different human melanomas was 98–116 μM 5min after administration. These levels decreased rapidly to approximately 1 μM at 480 min. PT levels in tumors were measured in parallel and also reached the highest concentration (28–33 μM) 5 min after administration, whereas the lowest concentration (~1 μM) was measured at 180 min. The half-life of PT in in vivo growing melanoma tumors was of 36–40 min [14]. These experimental data clearly exemplify the limitations of bioavailability that PT has (and polyphenols in general) and that temper its potential efficacy under in vivo conditions.

The topical administration of PT to cutaneous cancers, located in the skin and/or subcutaneous tissues, does not have the bioavailability problems mentioned above. Skin keratinocytes have different phase I (reduction/oxidation) and II (biotransformation) enzyme activities [17,18]. Accordingly, we found that topically administered PT (using liposomes as carriers) completely prevents chronic UVB-induced skin carcinogenesis [19]. This anticarcinogenic effect was associated with the maintenance of skin antioxidant defenses and the inhibition of UVB-induced oxidative damage [19]. Therefore, the anticancer activity of PT did not depend on direct antioxidant activity of the molecule itself.

## 3. Toxicity 

In Swiss mice fed with a PT-enriched diet at doses of 0, 30, 300, and 3000 mg/kg body weight/day red blood cell number and the hematocrit increased (approx. 25%) compared to control mice, whereas biochemical parameters were not significantly affected. Histopathology, hematology, clinical chemistry, and urinary balance studies found no alterations induced by PT as compared to controls [20], thus concluding that orally administered PT, even at the highest dose administered, was nontoxic. More recent studies suggest a no-observed-adverse-effect-level (NOAEL) of 200 mg of 3’-hydroxy-pterostilbene (a natural PT analog)/kg body weight/day in rats after oral administration [21].

At present, there are 13 registered clinical trials under www.clinicaltrials.gov (accessed on 14 February 2021) where PT has been used in humans, alone or in combination with other compounds. Based on the available data, orally administered PT appears safe at a dose of 125 mg twice daily (NCT01267227) [22]. Safety concerns forced a previous trial to stop, where the safety and activity of SRT501 (a formulation of resveratrol claimed to increase its in vivo bioavailability), alone or in combination with bortezomib, were being evaluated (NCT00920556). In this trial, where 5g of SRT501/day was administered orally for 20 consecutive days, kidney damage developed in some patients, thus raising the question of whether high doses (>1 g/day), administered chronically could pose toxicity problems. 

Intravenous administration has been also evaluated in nude mice. PT was found to be pharmacologically safe (no organ-specific or systemic toxicity, i.e., tissue histopathologic examination and regular hematology and clinical chemistry data) even when administered i.v. (dissolved in DMSO:ethanol [2:1]) at a dose of 30 mg/kg per day × 23 days [23], or at a dose of 40 mg/kg every 48h × 5 weeks [14]. The i.v. administration has never been tried in humans. However, the availability of a hydrosoluble disodium salt of PT phosphate (e.g., www.lgcstandards.com, accessed on 14 February 2021) may facilitate its use in cancer. 

## 4. Pharmaceutical Formulations, Structural Modifications, and Delivery Systems 

As it is in general the case for all natural polyphenols, the low systemic bioavailability of PT limits is anticancer potential. Consequently, methods to improve its absorption rate and pharmacokinetics, and thereby its bioavailability, would positively affect its therapeutic efficacy. On this issue and regarding polyphenols in general, different options have been recently discussed (see, e.g., Estrela 2017) [24]. Potential options to increase PT bioavailability may include:

(a) Prodrugs (i.e., carboxyesters, sulfonates, sulfates, phosphates, acetals, carbamates, and carbonates). The availability of a phosphorylated salt of PT, which increases its polarity and water solubility, has been mentioned above). Prodrugs in which the hydroxyl moiety is reversibly protected as a carbamate ester linked to the N-terminus of a natural amino acid (isoleucine or β-alanine) afforded increased absorption, reduced metabolism and higher concentrations of PT, sustained for several hours, in different organs [25]. Bis(hydroxymethyl)propionate analogs of PT have shown high anticancer activity against cisplatin-resistant human oral cancer cells [26].
(b) Solubilizing the compound in an organic solvent and subsequently adding it into an aqueous phase that contains a suitable stabilizer results in an emulsion. The homogenization of the emulsion and dilution in water may favor the precipitation of uniform nanoparticles. For instance, this methodology increases, e.g., curcumin bioavailability several fold [27]. In nanoemulsions, the higher unsaturation levels of lipids improved the lipid digestibility and PT bioaccessibility [28].
(c) Polyionic/polymeric shells encapsulating nanoparticles, solid lipid nanoparticles, or the conjugation of nanoparticles with ligands such as folic acid (which may recognize specific cell surface molecules in target cells), are additional options [29]. Moreover, an antibody-4arm-polyethylene glycol-PT conjugate has been synthesized for the targeted co-delivery of anticancer drugs to solid tumors [30]. Zein/fucoidan nanoparticles are a promising delivery carrier for the encapsulation, protection, and release of PT [31]. Whereas poly(2-oxazoline)-PT block copolymer nanoparticles, where a poly(2-methylsuccinate-2-oxazoline) segment conjugates PT, can be also used for dual anticancer drug delivery [32].
(d) Liposomes. For instance, lipophilic 3-oxo-C(12)-homoserine lactone and stilbene derivatives can be loaded into liposomal lipid bilayer with efficiencies of 50–70% [33]. Liposome-engulfed PT was highly efficient for the topical administration of PT [19]. Nevertheless, it has not been assayed for parenteral administration yet.
(e) Implantable drug delivery systems, such as Alzet-like reservoir pumps (ALZA/Durect Corp., Cupertino, CA) (controlled-release drug delivery which use osmotic gradients generated after their subcutaneous implantation); or matrix-type implants, which entrap the drug in a polymeric matrix and can provide high local concentrations of the drug and/or release it slowly into the blood circulation [34].
(f) Exosomes represent good vehicles due to their biocompatibility, stability in blood circulation, and even ability to target them to certain cell and tissue types. These carriers have already been used for other polyphenols [24], and its use has been suggested for PT [35].
(g) Cocrystals. The propensity of PT to form cocrystalline materials with active pharmaceutical ingredients was first studied by Schultheiss et al. [36], who found that the caffeine cocrystal solubility was 27× higher than the PT solubility. The same authors also reported the cocrystallization of PT with carbamazepine [37]. More recent advances are under development (e.g., www.circecrystal.com, accessed on 14 February 2021).

## 5. Anticancer Effects and Mechanisms 

In this section, we will summarize published reports that have examined the anticancer effects of PT under in vitro (Table 1) and in vivo (Table 2) conditions. In order for a publication to be included in Table 1, and reviewed here, a minimum of two cell lines needed to be examined unless PT was being examined in combination with another molecule. PT has garnered increasing interest from cancer researchers over the last decade as a natural molecule with anticancer properties that has been shown to be safe for human use (see above). 

### 5.1. Breast Cancer

The anticancer effects of PT were first examined against two breast cancer cell lines (MCF7 and ZR-751) in combination with tamoxifen [38]. These cells were treated with PT (10 and 20 μM) for 24 h prior to Tamoxifen (5 μM) being added and assayed for cell viability and apoptosis. This combination demonstrated the inhibition of cell proliferation and increased apoptosis in both cell lines. In addition, PT + Tamoxifen was shown to be additive in the reduction of cell viability in the ZR-751 cell line, but not the MCF7 cell line [38]. This finding is consistent with a subsequent study showing MCF7 to be resistant to PT anticancer activities due to high levels of HSP70 expression [39]. PT was then examined against the mutant p53-breast cancer cell lines MDA-MB-231 and T-47D, and found to decrease mutant p53 protein expression while increasing the expression of the pro-apoptotic Bax protein [40].

PT has been assessed in several mouse models of breast cancer as well. PT was shown to induce apoptosis in ER-α66 negative cells both in vitro and in a mouse xenograft model through the inhibition of Erk and Akt activation [41]. Specifically, oral PT (56 mg/kg) every four days for 3 weeks induced apoptosis and inhibited tumor growth. Interestingly, the authors show that knockdown of ER-α36 (a variant of ER-α66 present in this cell line) desensitized the cancer cells to PT [41].

PT was then examined for activity against the invasive ability of three breast cancer cell lines MCF7, Hs578t and MDA-MB-231 and found to inhibit the migratory potential of MDA-MB-231 and Hs578t cells [42]. Once again MCF7 cells proved resistant to PT treatment potentially due to high HSP70 expression [39]. Importantly, PT was shown to suppress tumor growth and metastasis in an MDA-MB-231 xenograft mouse model through reduction in src expression [42].

PT was then shown to inhibit tumor growth and invasion in combination with Vitamin E in a breast cancer xenograft model through inhibition of Akt and down regulation of cell cycle proteins [43]. In this study PT (40 μg/kg) was given orally three times per week while Vitamin E (42 and 99 IU/kg) were given in the diet daily. 

After demonstrating that PT exhibited the greatest dose-dependent antiproliferative activity against triple-negative MDA-MB-468 cells of the three different subtypes of breast cancer test in vitro, oral administration of PT was examined in an MDA-MB-468 xenograft mouse model [44]. PT (0.1% *w*/*w* in diet) suppressed tumor growth in this mouse model [44]. Investigation into the potential mechanism of PT antitumor activity using the MDA-MB-468 cell line revealed an inhibition of Akt and an upregulation of Bax protein [44]. 

### 5.2. Cervical Cancer

Efficacy of PT (20 and 40 μM) was examined in cervical cancer in both HeLa adherent and stem-like cells [45]. PT was found to inhibit growth, viability and migration of HeLa adherent cells through cell cycle arrest via induction of p53 coupled with reduction of cyclin E1 and cyclin B1 expression. PT also induced apoptosis through ROS-induced activation of caspase-3 and caspase-9 as well as downregulation of the antiapoptotic proteins Bcl-2 and Bcl-xL and the inhibition of MMP-2 and MMP-9 expression [45]. Furthermore, this was the first study to look the possible effect of PT on cervical cancer stem cell-like cells (CSCs). The authors report that PT suppressed tumor-sphere forming ability and migration of CSCs through reducing stemness supporting transcription factors (Sox2, Oct4 and Nanog) as well as decreasing the activation of STAT3 [45]. 

A separate study examined the effect of PT on HPV-E6 positive cervical cancer in vitro and in vivo [46]. PT was shown to be cytotoxic to E6-TC1 cells though decreasing the expression of E6 oncoprotein in vitro. A mouse xenograft of this cell line was then used to evaluate PT (1 mM) by intralesional injection daily for 5 days. In the model PT significantly reduced the size of the tumors by an average of 72% when compared to controls. Furthermore, immunohistochemistry of the PT-treated tumors depicted in increase in apoptosis through caspase-3 activation and reduced E6 expression as compared with control tumors [46].

### 5.3. Colon Cancer

The effects of PT in colon cancer were first assessed in 2008. A combination of PT with another polyphenol, quercetin was shown to inhibit the growth of HT-29 colorectal cancer cells by about 56% via an increase in SOD2 expression and a decrease in Bcl-2 expression [23]. Intravenous administration of this combination, PT and quercetin (each at 20 mg/kg/day), suppressed tumor growth by approximately 51% in HT-29 xenografts [23]. 

Another lab examined the role of PT alone in treating an azoxymethane (AOM)-induced colon cancer rat model [47]. PT was continuously administered orally via the diet at a concentration of 40 ppm for 45 weeks and resulted in reduced tumor multiplicity and downregulated the expression of proliferating cell nuclear antigen (PCNA), β-catenin and cyclin D1 [47].

An independent study of PT in the AOM-induced colon cancer model (in mice this time) once again showed that PT can inhibit AOM-induced colon tumorigenesis [48]. This study presented evidence that PT is working through reduction of NF-κB activation, decreasing the expression of inducible nitric oxide synthase (iNOS), cyclooxygenase 2 (COX-2) and aldose reductase as well as through activation of Nrf-2 [48].

### 5.4. Endometrial Cancer

Another investigation demonstrated that PT as a single treatment led to an increased cleavage of an apoptotic marker, caspase 3, and a decreased expression of cell survival proteins, Bcl-2 and Bcl-xL, in endometrial cells, similar to the pro-apoptotic effects by PT reported for other cancer cell lines [49]. In addition, PT inhibited the expression of the cell cycle regulators, such as cyclin D1, cyclin B1 and CDK4. These activities were further enhanced when PT was combined with megestrol acetate, a hormonal treatment in endometrial cancer, resulting in a synergistic antiproliferative effect in endometrial cancer cells. Investigation into molecular mechanisms leading to this synergy revealed that the combination more effectively suppressed the activation of the ERK1/2 pathway and STAT3, as well as ER expression. The combination of PT (30 mg/kg/day) and megestrol acetate (10 mg/kg/day) by oral gavage also significantly reduced tumor growth in a xenograft endometrial cancer mouse model, as demonstrated by reduction in tumor weight and volume [49]. 

An ongoing phase II randomized controlled, neoadjuvant trial for patients with endometrial cancer, currently investigates the in vivo effect of the combination of PT and megestrol acetate (Clinical Trials Identifier: NCT03671811). This is an open-label phase II randomized controlled trial of neoadjuvant therapy during the preoperative window period with megestrol acetate ± PT in patients with stage I endometrial cancer (EC). The objective is to evaluate the in vivo antiproliferative impact of PT in endometrial cancer, by the comparison of the Ki-67 proliferation index in post-treatment hysterectomy samples with pre-treatment endometrial samples. Other potentially predictive molecular and/or clinicopathologic biomarkers will be explored.

### 5.5. Ovarian Cancer

The anti-tumor activity of PT in human ovarian cancer was demonstrated in vitro. PT inhibited cell viability by suppressing cell cycle progression and inducing apoptosis. Additionally, it potentiated the anti-proliferative effects of cisplatin, a first line agent for ovarian cancer treatment. PT reduced proliferation and migration in ovarian cancer cells by the inhibition of the STAT3 pathway [50]. PT was found to inhibit STAT3 activation in OVCAR-8 and Caov-3 cells. STAT3 is constitutively activated in ovarian cancer and the inhibition of STAT3 pathway effectively suppresses ovarian cancer growth and progression. PT also caused the decreased expression of STAT3 target proteins, including anti-apoptotic proteins, such as Mcl-1 and Bcl-2 and cell cycle protein, such as cyclin D1. As a result, PT treatment leads to the induction of apoptosis and inhibition of cell cycle progression. 

The effect of PT on cell cycle progression appeared to be concentration dependent in both OVCAR-8 and Caov-3 cells. While a low concentration of PT (25 μM) led to an increase in cells in the S-phase, a higher concentration of PT (50–150 μM) caused an increase in cells in the G0/G1 phase. Consistent with these results, one of the critical proteins controlling cell cycle progression, cyclin D1, was remarkably downregulated by PT in a dose-dependent manner in both OVCAR-8 and Caov-3 cells. Therefore, it is possible that PT induced cell cycle arrest through the cyclin–CDK checkpoint.

### 5.6. Prostate Cancer

Initially, PT was examined in two prostate cancer cell lines for it anticancer activity, the p53 wild type LNCaP cells and the p53 null PC3 cell line [51]. This study demonstrated that PT exhibited a dose-dependent inhibition of cell proliferation regardless of p53 status through activation of AMPK. However, PT only induced apoptosis in the p53 null PC3 cells [51]. Another lab subsequently examined the ability of PT to regulate p53 activity in a prostate cancer xenograft model [52]. Intraperitoneal injection of PT (50 mg/kg/day) was reported to be effective in this model resulting in significantly inhibited tumor growth, progression, local invasion and metastasis. This reduction was reflective of decreased metastasis-associated protein 1 (MTA1) expression and subsequent decrease in p53 inactivation. In addition, the knockdown of metastasis-associated protein 1 (MTA1) further sensitized tumors to PT treatment [52]. 

This same lab reported on the ability of PT to regulate another tumor suppressor, PTEN, in prostate cancer pathology [53]. Building on a previous publication that elucidated the overexpression of the oncogenic microRNA-17 (miR-17) family, which downregulates PTEN in prostate cancer [54], this study focused on PT anticancer activity in a xenograft model of prostate cancer [53]. The authors show that I.P injection of PT (50 mg/kg) daily for 5 days/week for 39 days reduced tumor growth through the downregulation of miR-17-5p and miR-106-5p which rescued PTEN expression [53].

### 5.7. Pancreatic Cancer

The anticancer activities of PT in pancreatic cancer were first assessed in two cell lines, MIA PaCa and PANC-1 [55]. Treatment of these cells with PT (10 to 100 μM) showed a decrease in cell proliferation in a concentration- and time-dependent manner through the induction of cell cycle arrest, mitochondrial membrane depolarization and activation of caspases [55]. Another lab recently reported a similar finding that the treatment of PT (50 and 75 μM) inhibited cell proliferation of two pancreatic cell lines, MIA PaCa-2 and the gemcitabine-resistant MIA PaCa-2^GEMR^ [56]. PT was reported to induce cell cycle arrest in S-phase, increase apoptosis and inhibit the expression of the multidrug resistance protein (MDR1) in both cell lines. The reduction in MDR1 expression was due to decreased Akt activation [56]. 

Microarray analysis was performed on MIA PaCa-2 cells that were treated with PT (50 μM) to ascertain differential gene regulation of pancreatic cancer cells by PT [57]. This genetic analysis revealed that PT upregulated the pro-apoptotic genes including DDIT-3, growth differentiation factor 15 and SOD2. PT was then administered orally at one of three doses (100 μg/kg/day, 500 μg/kg/day or 1 mg/kg/day) to a MIA PaCa-2 xenograft mouse model. All doses of PT were found to inhibit tumor growth and tumor histology confirmed that all PT-treated tumors showed prominent central necrosis encompassing 60–80% of the tissue [57]. 

### 5.8. Skin Cancer

The anticancer activity of PT against skin cancer was first reported in 2005. The intravenous administration of PT (20 mg/kg/day) in combination with quercetin (20 mg/kg/day) was shown to be effective in a B16-F10 melanoma metastasis mouse model [16]. Specifically, PT + quercetin inhibited 73% of liver metastasis of B16-F10 cells. The observed antimetastatic activity was reported to be due to the inhibition of vascular adhesion molecule 1 expression in the hepatic sinusoidal epithelium thereby decreasing B16-F10 cell adhesion to the endothelium as well as down regulation of Bcl-2 in the metastatic cells [16]. Similarly, PT (10 to 50 μM) alone was shown to inhibit the cell proliferation and induce apoptosis of A375 melanoma cells, which have low HSP70 expression, in vitro [39]. 

PT prevented 7,12-dimethylbenz[a]anthracene (DMBA) + 12-O-tetradecanoylphorbol-13-acetate (TPA)-induced skin tumor formation via the downregulation of iNOS and COX-2 expression in mouse skin [58]. In this model, PT (1 or 5 μmol) or vehicle was applied topically 30 min prior to TPA application twice weekly for 20 weeks. After 20 weeks the control mice had an average of 38 skin tumors/mouse while the PT treated animals had significantly fewer tumors. The PT (1μmol) group had an average of 50% fewer tumors while the PT (5 μmol) group had a decrease in tumor number of about 63% [58].

Similar results were observed in a UVB-induced skin cancer mouse model. The topical administration of PT prevented chronic UVB (180 mJ/cm^2^, three times a week for 6 months)-induced carcinogenesis (90% of PT-treated mice did not develop skin carcinomas, whereas a large number of tumors were observed in all controls). In these experiments, 1–2 μmol PT/cm^2^ of skin were administered 20 min before each UVB irradiation [19]. The authors report that the anticancer activity of PT was attributed to Nrf2-dependent antioxidant response through the robust maintenance of glutathione levels as well as catalase, superoxide and GSH peroxidase activities [19].

The Nrf2 signaling pathway was examined further in mouse xenograft models of three melanoma cell lines, A2058, MeWo and MelJuso [14]. Interestingly, PT (15 μM) was not cytotoxic to any of these three cell lines in vitro, while i.v. PT (30 mg/kg) was efficacious against xenograft mouse models of these same three melanoma cell lines. Specifically, i.v. PT administered every 48 h for 5 weeks caused the significant inhibition of tumor growth of 70% in A2058, 65% in MeWo and 49% in MelJuso xenografted mice (Benlloch 2016). The mechanism of action proposed shows PT reduces circulating levels of adrenocorticotropin hormone (ACTH) resulting in a decrease in Nrf2-mediated antioxidant defenses in the melanoma cells. This mechanism does explain why PT was not effective against melanoma cells in culture and underscores the importance of testing potential anticancer agents under in vivo conditions. 

### 5.9. Lung Cancer

The most robust study of the anticancer activity of PT against lung cancer to date examined PT in combination with Osimertinib against five EGFR-mutation positive non-small cell lung cancer (NSCLC) cell lines. PT plus Osimertinib demonstrated synergistic effects on cell proliferation for all five cell lines tested [59]. Intriguingly, while PT alone did not have an effect on Src phosphorylation in these cells, PT did reverse the Osimertinib-induced STAT3, YAP1 and CUB domain containing protein (CDCP1) phosphorylation when treated in combination. If these results are replicated in vivo, the addition of PT could greatly improve the current monotherapy of Osimertinib in NSCLC since PT abrogates the known Osimertinib-activated resistance pathways [59]. In addition to this study, PT (10 to 50 μM) has been shown to effectively inhibit the growth of A549 lung cancer cells in vitro since this cell line has low HSP70 expression [39]. 

### 5.10. Liver Cancer

PT (100 and 200 mg/kg/day) via i.p. injection was shown to inhibit hepatocellular carcinoma (HCC) tumor growth in a chemically induced liver cancer mouse model [60]. In fact, a dose-dependent suppression of tumor growth was observed as there were both fewer tumors per mouse as well as reduced size of the tumors. The proposed mechanism for PT anticancer activity in liver cancer was assessed using HepG2 cells in vitro as well as in tumors and was attributed to PT-induced ROS generation through increased p53 expression which, in turn, decreased SOD2 expression [60]. 

### 5.11. Hematological Cancers 

Two robust studies have looked at the anticancer effects of PT in hematological cancers. The first examined PT against six diffuse large B-cell lymphoma (DLBCL) cell lines as well as i.v. PT (30 mg/kg) every two days for 20 days in a DLBCL xenograft mouse model [61]. PT (12.5–100 μM) exhibited a dose-dependent inhibition of cell viability in vitro through the reduction in mitochondrial membrane potential, increased ROS generation and increased apoptosis via caspase activation. Similarly, PT administered i.v. significantly inhibited DLBCL tumor growth in mice [61]. The second study tested the potential anticancer activity of PT against multiple myeloma (MM) cell lines in a xenograft mouse model. Specifically, PT (10-50 μM) demonstrated a dose-dependent reduction in cell proliferation on four MM cell lines [62]. The observed cytotoxic effect was due to increased caspase activation specifically in MM cells and not in normal peripheral blood mononuclear cells. PT also increased ROS generation and reduced mitochondrial membrane potential. Additionally, i.p. injection of PT (50 mg/kg) daily for two weeks inhibited MM tumor growth in a mouse xenograft model [62]. All of these findings echo the anticancer activity of PT in DLBCL [61]. 

In addition, it has been proposed the mechanism of action to be ERK1/2 and JNK activation [62]. This seems counterintuitive on its own as ERK1/2 activation has been shown to stimulate Nrf2 nuclear localization and induce antioxidant defenses [63], however, since tumor growth was clearly reduced after PT treatment, it is entirely possible that PT inhibits AKT in MM and thereby inhibits Nrf2 activation as evidenced through enhanced ROS generation. Although AKT activation was not assessed in this study, AKT inhibition by PT has been shown in pancreatic [56] and breast cancer [41,43,44]. Furthermore, the inhibition of mTOR (though the inhibition of AKT) has been proposed as a potential adjunct therapy in MM [64]. The authors of this manuscript opine that the use of an inhibitor of mTOR complex 2, via the inhibition of AKT, in combination with an inhibitor of mTOR complex 1 in multiple myeloma would be useful for the anti-angiogenic management of patients [64]. Thus, a potential clinical study is warranted using PT as an mTOR complex 1 inhibitor with a TBD mTOR complex 2 inhibitor. 5.12. Other Cancers (Oral, Gastrointestinal, Biliary, Glioblastoma Multiforme)

The anticancer activity of PT was examined in a cisplatin-resistant human oral cancer CAR cells in vitro. PT (50 μM and 75 μM) treatment of these cells reduced cell viability though the induction of apoptotic caspase activation and the downregulation of MDR1 through reduced Akt activation [65]. PT was also shown to inhibit the cell proliferation of three gastrointestinal cancer cell lines in vitro [66]. Once again, PT (10 and 100 μM) treatment was found to increase mitochondrial membrane potential and increase ROS generation, leading to increased apoptosis [66]. 

Instead of inducing apoptosis in human cholangiocarcinoma (CCA), a cancer of the biliary tract, PT (15–120 μM) treatment in vitro of two CCA cell lines showed dose-dependent cytotoxic effects through autophagy [67]. This study also demonstrated that PT (30 mg/kg and 60 mg/kg) administered i.p. every two days for three weeks inhibited CCA tumor growth in a mouse xenograft model [67]. 

Another lab targeting glioblastoma multiforme (GBM) examined PT anticancer activity against glioma stem cells (GSCs) since they have been shown to contribute to the tumorigenesis, recurrence and resistance to treatment [68]. They showed that PT suppressed self-renewal and irradiation resistant properties of GSCs and this activity was associated with miR-205 increased expression. This microRNA downregulates GRP78, a protein that is highly expressed in GSCs and contributes to treatment resistance. PT also suppressed tumorigenesis in GSC xenografted mice [68].

### 5.12. Summary of Proposed Anticancer Activities of Pterostilbene

**Figure 1:**
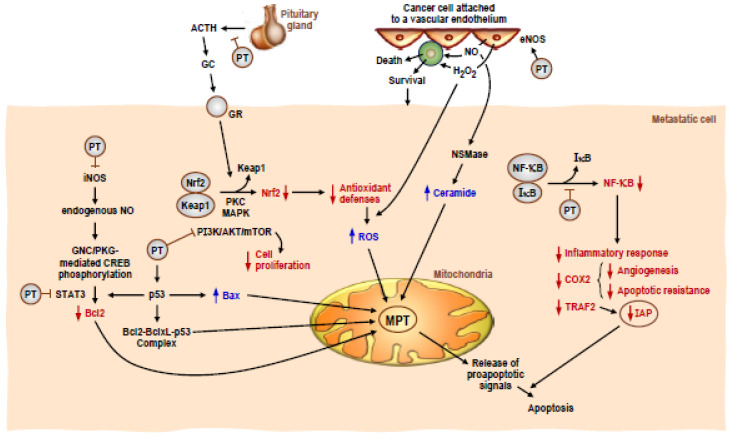
Potential molecular mechanisms involved in pterostilbene-induced cancer cell death. The multiple molecular interactions and signaling mechanisms are based on or deduced from data obtained in metastatic melanoma cells under in vivo conditions. Pterostilbene (PT) is encircled, and its interactions (inhibitions or activations are indicated by a T line or an arrow, respectively). Abbreviations: iNOS, inducible nitric oxide synthase; nitric oxide, NO; guanylate cyclase, GNC; CAMP responsive element binding protein, CREB; signal transducer and activator of transcription 3, STAT3; B-cell lymphoma 2, Bcl2; B-cell lymphoma-extra large, BclxL; tumor protein p53, p53; Bcl2-assciated X protein, Bax; adrenocorticotropic hormone, ACTH; glucocorticoid, GC; glucocorticoid receptor, GR; nuclear factor erythroid 2-related factor 2, Nrf2; Kelch-like ECH-associated protein 1, Keap1; protein kinase C PKC; mitogen-activated protein kinase, MAPK; phosphoinositide 3 kinase/protein kinase B/mechanistic target of rapamycin, PI3/AKT/mTOR; endothelial nitric oxide synthase, eNOS; reactive oxygen species, ROS; neutral sphingomyelinase, NSMase; mitochondrial permeability transition, MPT; nuclear factor kappa-light-chain-enhancer of activated B cells, NF-kB; nuclear factor of kappa light polypeptide gene enhancer in B-cells inhibitor, IkB; cyclooxygenase 2, COX2; TNF receptor-associated factor 2, TRAF2; inhibitors of apoptosis proteins, IAP.

As this review illustrates, the anticancer activity of PT has been well documented in a wide range of cancers over the last 16 years. While published accounts of the mechanism of action of PT against cancer cells can sometimes look contradictory, depending on the experimental model, concentrations used, time of exposure and route of administration, it is clear that PT possesses several different mechanisms of anticancer action in its repertoire. Figure 1 depicts several distinct anticancer activities of PT highlighted in this review. Specifically, PT has been shown to downregulate iNOS in both skin [58] and colon cancer [48] models, which leads to the increased apoptosis of the cancer cells. Along the same downstream pathway, PT has been shown to inhibit STAT3 in lung [59], ovarian [50], pancreatic [57] and endometrial cancer [49]. PT can also function through inhibiting the AKT/mTOR pathway in pancreatic [56] and breast cancers [41,43,44]. Furthermore, PT can exhibit anticancer activity through the inhibition of NF-κB signaling in colon [48] and skin cancer [58]. Finally, Figure 1 shows an anticancer activity of PT that only works in vivo, when PT indirectly downregulates Nrf2 through the inhibition of glucocorticoid secretion from the pituitary gland, which decreases antioxidant defenses of metastatic melanoma cells and leads to apoptosis [14] (see also [69]). The Benlloch et al. paper [14] clearly demonstrates the importance of using in vivo experimental models to examine the anticancer activity of PT as PT has indirect as well as direct effects on inducing cancer cell death. 

Another common theme highlighted here is that PT is an excellent candidate for adjunct therapy due to its multiple mechanisms of action. This is evidenced by PT reversing Osimertinib-induced STAT3 activation in lung cancer [59], PT inducing cell cycle arrest in gemcitabine-resistant pancreatic cancer cells [56], PT demonstrating synergistic efficacy with cisplatin in ovarian cancer cells [50], PT induction of apoptosis in cisplatin-resistance oral cancer cells [65], and PT showing an additive effect with tamoxifen in breast cancer cells [38]. In addition, PT was shown to synergize with megestrol acetate for the reduction in endometrial tumors in a xenograph model [49]. In fact, this study led to the first human clinical study to assess the anticancer effects of PT. An ongoing phase II randomized controlled, neoadjuvant trial for patients with endometrial cancer, investigating the effect of the combination of PT and megestrol acetate (clinical trial identifier: NCT03671811).

**Table 1:** PT and cancer cells under in vitro conditions: effects and proposed mechanisms. NSCLC: non-small cell lung cancer.

Cancer Type	Concentration(s) Analyzed	Time of Incubation (hours)	Anticancer Effect	Proposed Mechanism	Reference
Lung	PT (10 μM) + Osimertinib (0.02 μM)	24	Synergistic anticancer effect against two EGFR-mutation positive NSCLC cells	The combination reversed osimertinib-induced STAT3 activation and suppressed src activation	[59]
Cervical	PT (20 and 40 μM)	48	Inhibition of growth and metastatic ability of both adherent and stem-like cancer cells	Induction of ROS-induced apoptosis and inhibition of MMP 2/9 expression	[45]
Pancreatic	PT (50 and 75 μM)	72	Induced cell cycle arrest and apoptosis in Gemcitabine-resistant cancer cells	Inhibitions of multidrug resistance protein (MDR1) expression via reduction in Akt signaling	[56]
Ovarian	PT (18.5 to 300 μM) +/− Cisplatin (3.125 to 50 μM)	48	Induction of cell cycle arrest and apoptosis against several ovarian cancer cell lines and synergy with cisplatin	Downregulation of JAK/STAT3 pathway	[50]
Oral	PT (50 and 75 μM)	24 or 48	Induction of apoptosis of cisplatin-resistant oral cancer cells	Activation of intrinsic apoptosis cascade and downregulation of MDR1	[65]
Breast	PT (2.5 to 10 μM)	24	Upregulation of apoptotic pathways in two mutant-p53 cell lines	Induction of pro-apoptotic Bax protein and caspase-3 activity. Decreased mutant p53 protein	[40]
Breast	PT (10 and 20 μM) + Tamoxifen (5 μM)	24	PT + Tamoxifen showed an additive inhibitory effect on breast cancer cells	Increased apoptosis	[38]
Gastrointestinal	PT (10 and 100 μM)	48	PT showed dose-dependent inhibition of cell proliferation in three GI cancer cell lines	Increase in mitochondrial membrane potential, ROS and lipid peroxide	[66]
Prostate	PT (10 to 100 μM)	48	PT showed dose-dependent inhibition of cellular proliferation in three prostate cancer cell lines	Activation of AMPK	[51]
Pancreatic	PT (10 to 100 μM)	72	PT is cytotoxic against two pancreatic cancer cell lines.	Inhibition of cell proliferation and/or cell death, mitochondrial membrane depolarization and activation of caspases.	[55]
Melanoma, colon, breast, and lung	PT (10 to 50 μM)	72	PT demonstrates differential toxicity to various cancer cell lines	PT is more efficacious in melanoma and lung cancer cells that have low HSP70 expression than in high HSP70 colon and breast cancer cells	[39]

**Table 2:** PT and cancer: in vivo evidences. AOM: azoxymethane; HCC: hepatocellular carcinoma; TPA: 12-O-tetradecanoylphorbol-13-acetate.

Cancer Type	Concentration(s) Analyzed	Administration	Anticancer Effect	Proposed Mechanism	Reference
Cervical	PT (1 mM)	Intralesional injection daily for 5 days	PT inhibits tumor development in HPV E6-positive cervical cancer mouse model	Decrease in tumor size due to increase in apoptosis, and downregulation of E6 and VEGF tumor protein levels	[46]
Breast	PT (40 μg/kg) + Vitamin E (42 IU/kg or 99 IU/kg)	PT oral 3 times per week Vit E in diet	PT and vit E inhibited breast tumor growth and invasion in mouse xenograft model	Inhibition of Akt and downregulation of cell cycle proteins	[43]
Breast	PT (56 mg/kg every 4 days for 3 weeks)	Oral gavage	PT induces apoptosis and inhibits tumor growth of ER- Breast cancer xenograft model	Inhibition of ER-a36 (a variant of full-length Estrogen receptor) resulting in inhibition of Akt signaling	[41]
Prostate	PT (50 mg/kg)	Intraperitoneal Injections daily (5 days/week) for 39 days	PT reduced tumor growth in mouse xenograft model	Downregulation of miR-17-5p and miR-106-5p expression in both tumors and circulation	[53]
Breast	PT (10 mg/kg)	Intraperitoneal injections 3 times a week	PT suppressed tumor growth and metastasis in xenograft mouse model	Reduction in src signaling and inhibition of EMT	[42]
Pancreatic	PT (100 μg/kg, 500 μg/kg or 1 mg/kg)	Oral gavage	PT inhibited tumor growth rates	Increases MnSOD antioxidant activity; inhibits STAT3 activity	[57]
Melanoma	PT (30 mg/kg) every 48 h for 5 weeks	Intravenous	PT decreased tumor growth in mouse xenograft model	Downregulated adrenocorticotropin hormone (ACTH) resulting in decrease Nrf2-mediated antioxidant defenses	[14]
Lymphoma	PT (30 mg/kg every 2 days for 20 days)	Intravenous	PT inhibited tumor growth in diffuse large B-cell lymphoma xenograft mouse model	Cytotoxic effect due to reduction in mitochondrial membrane potential and increase in apoptosis and ROS levels	[61]
Breast	PT (0.1% *w*/*w* in diet)	Oral	PT suppressed tumor growth in triple-negative breast cancer xenograft mouse model	Inhibition of Akt activationand upregulation of Bax	[44]
Prostate	PT (50 mg/kg/day)	Intraperitoneal	PT inhibited tumor growth and metastasis in prostate cancer xenografts	Reduction in metastasis-associated protein 1 (MTA1) and increased apoptosis	[52]
Endometrial	PT (30 mg/kg/day) + Megestrol acetate (10 mg/kg/day)	Oral gavage	PT synergizes with megestrol acetate for reduction of tumor growth in xenografts	Suppression of STAT3 activation as well as decreased ER expression	[49]
Biliary	PT (30 and 60 mg/kg every 2 days For 3 weeks)	Intraperitoneal	PT inhibited tumor growth in xenograft mouse model	Inhibited cell progression and induced autophagy	[67]
Multiple Myeloma	PT (50 mg/kg/day For 2 weeks)	Intraperitoneal	PT reduced tumor volume in mouse xenografts	Inhibited cell progression. Induction of apoptosis through increased ROS generation and activation of ERK1/2 and of JNK signaling	[62]
Colon	PT (40 ppm diet for 45 weeks)	Oral	PT reduced AOM-induced colon tumor multiplicity	Inhibits cell proliferation via reduced PCNA expression and reduced beta-catenin and cyclin D1. Reduction of inflammatory markers	[47]
Colorectal	PT (20 mg/kg/day) + quercetin (20 mg/kg/day)	Intravenous	PT + QUER inhibited tumor growth by 51% in xenografts	Increase in SOD2 expression and decrease in Bcl-2 expression	[23]
Liver	PT (100 and 200 mg/kg/day)	Intraperitoneal	PT dose-dependently inhibited HCC tumor growth in mouse model	Increase in p53 expression and ROS generation and activation of apoptosis	[60]
Skin	PT (1-2 μmol)	Topical	PT prevented UV-B induced skin cancer in mouse model	Maintenance of skin antioxidant defenses including Nrf2 activation	[19]
Skin	PT (1 and 5 μmol)	Topical	PT suppressed TPA-induced skin cancer in mouse model	Downregulation of iNOS and COX-2	[58]
Glioblastoma Multiforme	PT (2 mg/kg, three times a week)	Intraperitoneal	PT suppressed tumorigenesis in glioma stem cell mouse xenograft	Inhibition of GRP78	[68]
Colon	PT (50 and 250 ppm in diet, 24 weeks)	Oral	PT prevents AOM-induced colon tumorigenesis.	Reduction of NF-κB activation, as well as iNOS and COX-2 expression Activation of Nrf2 signaling	[48]
Melanoma	PT (20 mg/kg/day) + QUER (20 mg/kg/day)	Intravenous	PT + QUER shown to inhibit metastasis of melanoma in xenografts	Inhibition of Bcl-2	[16]

## 6. Concluding Remarks JME 

Accumulated experimental evidence suggest that PT exhibits the hallmark characteristics of an effective anticancer agent based on its antineoplastic properties in different cancers. Its molecular structure favors a higher bioavailability compared to that showed by resveratrol. Importantly, PT stands out (as compared to almost all other natural polyphenols) in its ability to cross the blood–brain barrier, which may confer the potential advantage of being useful in the therapy of tumors of the central nervous system, such as glioblastoma multiforme. Nevertheless, reaching effective concentrations in tumors, under in vivo conditions, and for a sufficient time to exert its antineoplastic actions, is a fundamental unsolved problem. A key problem directly related to this is its short half-life and low bioavailability under in vivo conditions. In fact, the most common discrepancy between experimental and clinical observations is the use of nonphysiologically relevant concentrations of the stilbene in mechanistic studies. Thus, it remains highly controversial how applicable underlying mechanisms are with bioavailable concentrations and biological half-life. In fact, the assumption that PT-induced anticancer effects derive from its direct interaction with the tumor cells frequently ignores the limitations involving in vivo concentrations and time of exposure. This reasoning leads to some obvious needs for future developments, i.e., (a) improvement of pharmaceutical formulations and delivery systems to increase bioavailability, (b) PT has chemo- and radiosensitizing effects which make it suitable as adjuvant in oncotherapy, (c) its very low systemic toxicity is a clear advantage for its combination with conventional/targeted anticancer treatments, (d) the combination of PT with other polyphenols may show synergic/additive effects. However, we still need more studies to show that these technological advances impact on improvements in the in vivo anti-cancer efficacy of the stilbene. Moreover, advances in experimental models still must be applied to humans. At present, there is only one clinical trial (phase 2) running where PT is used against a specific type of cancer (NCT03671811, Megestrol Acetate with or without PT in Treating Patients with Endometrial Cancer Undergoing Hysterectomy). In this regard, experimental evidences suggest the potential of combining PT with, e.g., prooxidant and/or anti-inflammatory strategies, anti-Bcl-2 therapies, HSP70 inhibitors, or PI3K/AKT/mTOR inhibitors. Besides, the authors of this review are aware that new advances are underway aiming to increase the anticancer efficacy of PT. 

PT is a magnificent example of how a molecule, originally produced in nature for the defense of plants, can be developed until its application in human oncotherapy.

## References

[1] Kim H., Seo K.-H., Yokoyama W. (2020). Chemistry of Pterostilbene and Its Metabolic Effects. J. Agric. Food Chem..

[2] Spath E., Schlager J. (1940). On the constituents of “Red Sandalwood” [Pterocarpus santalinus]. 2: The constitution of pterostilbene. Ber. Dtsch. Chem. Gesellsch..

[3] Tsai H.-Y., Ho C.-T., Chen Y.-K. (2017). Biological Actions and Molecular Effects of Resveratrol, Pterostilbene, and 3’-Hydroxypterostilbene. J. Food Drug Anal..

[4] Schmidlin L., Poutaraud A., Claudel P., Mestre P., Prado E., Santos-Rosa M., Wiedemann-Merdinoglu S., Karst F., Merdinoglu D., Hugueney P. (2008). A Stress-Inducible Resveratrol O-Methyltransferase Involved in the Biosynthesis of Pterostilbene in Grapevine. Plant Physiol..

[5] Chong J., Poutaraud A., Hugueney P. (2009). Metabolism and Roles of Stilbenes in Plants. Plant Sci..

[6] Quideau S., Deffieux D., Douat-Casassus C., Pouységu L. (2011). Plant Polyphenols: Chemical Properties, Biological Activities, and Synthesis. Angew. Chem. Int. Ed. Engl..

[7] Estrela J.M., Ortega A., Mena S., Rodriguez M.L., Asensi M. (2013). Pterostilbene: Biomedical Applications. Crit. Rev. Clin. Lab. Sci..

[8] Liu Y., You Y., Lu J., Chen X., Yang Z. (2020). Recent Advances in Synthesis, Bioactivity, and Pharmacokinetics of Pterostilbene, an Important Analog of Resveratrol. Molecules.

[9] Szatrowski T.P., Nathan C.F. (1991). Production of Large Amounts of Hydrogen Peroxide by Human Tumor Cells. Cancer Res..

[10] Ortega A.L., Mena S., Estrela J.M. (2010). Oxidative and Nitrosative Stress in the Metastatic Microenvironment. Cancers.

[11] Gill J.G., Piskounova E., Morrison S.J. (2016). Cancer, Oxidative Stress, and Metastasis. Cold Spring Harb. Symp. Quant. Biol..

[12] Galadari S., Rahman A., Pallichankandy S., Thayyullathil F. (2017). Reactive Oxygen Species and Cancer Paradox: To Promote or to Suppress?. Free Radic. Biol. Med..

[13] Gorrini C., Harris I.S., Mak T.W. (2013). Modulation of Oxidative Stress as an Anticancer Strategy. Nat. Rev. Drug Discov..

[14] Benlloch M., Obrador E., Valles S.L., Rodriguez M.L., Sirerol J.A., Alcácer J., Pellicer J.A., Salvador R., Cerdá C., Sáez G.T. (2016). Pterostilbene Decreases the Antioxidant Defenses of Aggressive Cancer Cells In Vivo: A Physiological Glucocorticoids- and Nrf2-Dependent Mechanism. Antioxid. Redox Signal..

[15] Okazaki K., Papagiannakopoulos T., Motohashi H. (2020). Metabolic Features of Cancer Cells in NRF2 Addiction Status. Biophys. Rev..

[16] Ferrer P., Asensi M., Segarra R., Ortega A., Benlloch M., Obrador E., Varea M.T., Asensio G., Jordá L., Estrela J.M. (2005). Association between Pterostilbene and Quercetin Inhibits Metastatic Activity of B16 Melanoma. Neoplasia.

[17] Strohm B.H., Kulkarni A.P. (1986). Peroxidase, an Alternate Pathway to Cytochrome P-450 for Xenobiotic Metabolism in Skin: Partial Purification and Properties of the Enzyme from Neonatal Rat Skin. J. Biochem. Toxicol..

[18] Korkina L., De Luca C., Pastore S. (2012). Plant Polyphenols and Human Skin: Friends or Foes. Ann. N. Y. Acad. Sci..

[19] Sirerol J.A., Feddi F., Mena S., Rodriguez M.L., Sirera P., Aupí M., Pérez S., Asensi M., Ortega A., Estrela J.M. (2015). Topical Treatment with Pterostilbene, a Natural Phytoalexin, Effectively Protects Hairless Mice against UVB Radiation-Induced Skin Damage and Carcinogenesis. Free Radic. Biol. Med..

[20] Ruiz M.J., Fernández M., Picó Y., Mañes J., Asensi M., Carda C., Asensio G., Estrela J.M. (2009). Dietary Administration of High Doses of Pterostilbene and Quercetin to Mice Is Not Toxic. J. Agric. Food Chem..

[21] Majeed M., Bani S., Natarajan S., Pandey A., Naveed S. (2017). Evaluation of 90 Day Repeated Dose Oral Toxicity and Reproductive/Developmental Toxicity of 3’-Hydroxypterostilbene in Experimental Animals. PLoS ONE.

[22] Riche D.M., McEwen C.L., Riche K.D., Sherman J.J., Wofford M.R., Deschamp D., Griswold M. (2013). Analysis of Safety from a Human Clinical Trial with Pterostilbene. J. Toxicol..

[23] Priego S., Feddi F., Ferrer P., Mena S., Benlloch M., Ortega A., Carretero J., Obrador E., Asensi M., Estrela J.M. (2008). Natural Polyphenols Facilitate Elimination of HT-29 Colorectal Cancer Xenografts by Chemoradiotherapy: A Bcl-2- and Superoxide Dismutase 2-Dependent Mechanism. Mol. Cancer Ther..

[24] Estrela J.M., Mena S., Obrador E., Benlloch M., Castellano G., Salvador R., Dellinger R.W. (2017). Polyphenolic Phytochemicals in Cancer Prevention and Therapy: Bioavailability versus Bioefficacy. J. Med. Chem..

[25] Azzolini M., Mattarei A., La Spina M., Fanin M., Chiodarelli G., Romio M., Zoratti M., Paradisi C., Biasutto L. (2017). New Natural Amino Acid-Bearing Prodrugs Boost Pterostilbene’s Oral Pharmacokinetic and Distribution Profile. Eur. J. Pharm. Biopharm..

[26] Hsieh M.-T., Huang L.-J., Wu T.-S., Lin H.-Y., Morris-Natschke S.L., Lee K.-H., Kuo S.-C. (2018). Synthesis and Antitumor Activity of Bis(Hydroxymethyl)Propionate Analogs of Pterostilbene in Cisplatin-Resistant Human Oral Cancer Cells. Bioorg. Med. Chem..

[27] Shaikh J., Ankola D.D., Beniwal V., Singh D., Kumar M.N.V.R. (2009). Nanoparticle Encapsulation Improves Oral Bioavailability of Curcumin by at Least 9-Fold When Compared to Curcumin Administered with Piperine as Absorption Enhancer. Eur. J. Pharm. Sci..

[28] Liu Q., Chen J., Qin Y., Jiang B., Zhang T. (2019). Encapsulation of Pterostilbene in Nanoemulsions: Influence of Lipid Composition on Physical Stability, in Vitro Digestion, Bioaccessibility, and Caco-2 Cell Monolayer Permeability. Food Funct..

[29] Bansal S.S., Goel M., Aqil F., Vadhanam M.V., Gupta R.C. (2011). Advanced Drug Delivery Systems of Curcumin for Cancer Chemoprevention. Cancer Prev. Res. Phila..

[30] Liu K.-F., Liu Y.-X., Dai L., Li C.-X., Wang L., Liu J., Lei J.-D. (2018). A Novel Self-Assembled PH-Sensitive Targeted Nanoparticle Platform Based on Antibody-4arm-Polyethylene Glycol-Pterostilbene Conjugates for Co-Delivery of Anticancer Drugs. J. Mater. Chem. B.

[31] Liu Q., Chen J., Qin Y., Jiang B., Zhang T. (2020). Zein/Fucoidan-Based Composite Nanoparticles for the Encapsulation of Pterostilbene: Preparation, Characterization, Physicochemical Stability, and Formation Mechanism. Int. J. Biol. Macromol..

[32] Romio M., Morgese G., Trachsel L., Babity S., Paradisi C., Brambilla D., Benetti E.M. (2018). Poly(2-Oxazoline)-Pterostilbene Block Copolymer Nanoparticles for Dual-Anticancer Drug Delivery. Biomacromolecules.

[33] Coimbra M., Isacchi B., van Bloois L., Torano J.S., Ket A., Wu X., Broere F., Metselaar J.M., Rijcken C.J.F., Storm G. (2011). Improving Solubility and Chemical Stability of Natural Compounds for Medicinal Use by Incorporation into Liposomes. Int. J. Pharm..

[34] Liechty W.B., Peppas N.A. (2012). Expert Opinion: Responsive Polymer Nanoparticles in Cancer Therapy. Eur. J. Pharm. Biopharm..

[35] Mak K.-K., Wu A.T.H., Lee W.-H., Chang T.-C., Chiou J.-F., Wang L.-S., Wu C.-H., Huang C.-Y.F., Shieh Y.-S., Chao T.-Y. (2013). Pterostilbene, a Bioactive Component of Blueberries, Suppresses the Generation of Breast Cancer Stem Cells within Tumor Microenvironment and Metastasis via Modulating NF-ΚB/MicroRNA 448 Circuit. Mol. Nutr. Food Res..

[36] Schultheiss N., Bethune S., Henck J.-O. (2010). Nutraceutical Cocrystals: Utilizing Pterostilbene as a Cocrystal Former. CrystEngComm.

[37] Bethune S.J., Schultheiss N., Henck J.-O. (2011). Improving the Poor Aqueous Solubility of Nutraceutical Compound Pterostilbene through Cocrystal Formation. Cryst. Growth Des..

[38] Mannal P., McDonald D., McFadden D. (2010). Pterostilbene and Tamoxifen Show an Additive Effect against Breast Cancer in Vitro. Am. J. Surg..

[39] Mena S., Rodríguez M.L., Ponsoda X., Estrela J.M., Jäättela M., Ortega A.L. (2012). Pterostilbene-Induced Tumor Cytotoxicity: A Lysosomal Membrane Permeabilization-Dependent Mechanism. PLoS ONE.

[40] Elsherbini A.M., Sheweita S.A., Sultan A.S. (2020). Pterostilbene as a Phytochemical Compound Induces Signaling Pathways Involved in the Apoptosis and Death of Mutant P53-Breast Cancer Cell Lines. Nutr. Cancer.

[41] Pan C., Hu Y., Li J., Wang Z., Huang J., Zhang S., Ding L. (2014). Estrogen Receptor-A36 Is Involved in Pterostilbene-Induced Apoptosis and Anti-Proliferation in in Vitro and in Vivo Breast Cancer. PLoS ONE.

[42] Su C.-M., Lee W.-H., Wu A.T.H., Lin Y.-K., Wang L.-S., Wu C.-H., Yeh C.-T. (2015). Pterostilbene Inhibits Triple-Negative Breast Cancer Metastasis via Inducing MicroRNA-205 Expression and Negatively Modulates Epithelial-to-Mesenchymal Transition. J. Nutr. Biochem..

[43] Tam K.-W., Ho C.-T., Tu S.-H., Lee W.-J., Huang C.-S., Chen C.-S., Wu C.-H., Lee C.-H., Ho Y.-S. (2018). α-Tocopherol Succinate Enhances Pterostilbene Anti-Tumor Activity in Human Breast Cancer Cells in Vivo and in Vitro. Oncotarget.

[44] Wakimoto R., Ono M., Takeshima M., Higuchi T., Nakano S. (2017). Differential Anticancer Activity of Pterostilbene against Three Subtypes of Human Breast Cancer Cells. Anticancer Res..

[45] Shin H.J., Han J.M., Choi Y.S., Jung H.J. (2020). Pterostilbene Suppresses Both Cancer Cells and Cancer Stem-Like Cells in Cervical Cancer with Superior Bioavailability to Resveratrol. Molecules.

[46] Chatterjee K., Mukherjee S., Vanmanen J., Banerjee P., Fata J.E. (2019). Dietary Polyphenols, Resveratrol and Pterostilbene Exhibit Antitumor Activity on an HPV E6-Positive Cervical Cancer Model: An in Vitro and in Vivo Analysis. Front. Oncol..

[47] Paul S., DeCastro A.J., Lee H.J., Smolarek A.K., So J.Y., Simi B., Wang C.X., Zhou R., Rimando A.M., Suh N. (2010). Dietary Intake of Pterostilbene, a Constituent of Blueberries, Inhibits the Beta-Catenin/P65 Downstream Signaling Pathway and Colon Carcinogenesis in Rats. Carcinogenesis.

[48] Chiou Y.-S., Tsai M.-L., Nagabhushanam K., Wang Y.-J., Wu C.-H., Ho C.-T., Pan M.-H. (2011). Pterostilbene Is More Potent than Resveratrol in Preventing Azoxymethane (AOM)-Induced Colon Tumorigenesis via Activation of the NF-E2-Related Factor 2 (Nrf2)-Mediated Antioxidant Signaling Pathway. J. Agric. Food Chem..

[49] Wen W., Lowe G., Roberts C.M., Finlay J., Han E.S., Glackin C.A., Dellinger T.H. (2017). Pterostilbene, a Natural Phenolic Compound, Synergizes the Antineoplastic Effects of Megestrol Acetate in Endometrial Cancer. Sci. Rep..

[50] Wen W., Lowe G., Roberts C.M., Finlay J., Han E.S., Glackin C.A., Dellinger T.H. (2018). Pterostilbene Suppresses Ovarian Cancer Growth via Induction of Apoptosis and Blockade of Cell Cycle Progression Involving Inhibition of the STAT3 Pathway. Int. J. Mol. Sci..

[51] Lin V.C.-H., Tsai Y.-C., Lin J.-N., Fan L.-L., Pan M.-H., Ho C.-T., Wu J.-Y., Way T.-D. (2012). Activation of AMPK by Pterostilbene Suppresses Lipogenesis and Cell-Cycle Progression in P53 Positive and Negative Human Prostate Cancer Cells. J. Agric. Food Chem..

[52] Li K., Dias S.J., Rimando A.M., Dhar S., Mizuno C.S., Penman A.D., Lewin J.R., Levenson A.S. (2013). Pterostilbene Acts through Metastasis-Associated Protein 1 to Inhibit Tumor Growth, Progression and Metastasis in Prostate Cancer. PLoS ONE.

[53] Dhar S., Kumar A., Rimando A.M., Zhang X., Levenson A.S. (2015). Resveratrol and Pterostilbene Epigenetically Restore PTEN Expression by Targeting OncomiRs of the MiR-17 Family in Prostate Cancer. Oncotarget.

[54] Kumar A., Rimando A.M., Levenson A.S. (2017). Resveratrol and Pterostilbene as a MicroRNA-Mediated Chemopreventive and Therapeutic Strategy in Prostate Cancer. Ann. N. Y. Acad. Sci..

[55] Mannal P.W., Alosi J.A., Schneider J.G., McDonald D.E., McFadden D.W. (2010). Pterostilbene Inhibits Pancreatic Cancer in Vitro. J. Gastrointest. Surg..

[56] Hsu Y.-H., Chen S.-Y., Wang S.-Y., Lin J.-A., Yen G.-C. (2020). Pterostilbene Enhances Cytotoxicity and Chemosensitivity in Human Pancreatic Cancer Cells. Biomolecules.

[57] McCormack D.E., Mannal P., McDonald D., Tighe S., Hanson J., McFadden D. (2012). Genomic Analysis of Pterostilbene Predicts Its Antiproliferative Effects against Pancreatic Cancer in Vitro and in Vivo. J. Gastrointest. Surg..

[58] Tsai M.-L., Lai C.-S., Chang Y.-H., Chen W.-J., Ho C.-T., Pan M.-H. (2012). Pterostilbene, a Natural Analogue of Resveratrol, Potently Inhibits 7,12-Dimethylbenz[a]Anthracene (DMBA)/12-O-Tetradecanoylphorbol-13-Acetate (TPA)-Induced Mouse Skin Carcinogenesis. Food Funct..

[59] Bracht J.W.P., Karachaliou N., Berenguer J., Pedraz-Valdunciel C., Filipska M., Codony-Servat C., Codony-Servat J., Rosell R. (2019). Osimertinib and Pterostilbene in EGFR-Mutation-Positive Non-Small Cell Lung Cancer (NSCLC). Int. J. Biol. Sci..

[60] Guo L., Tan K., Wang H., Zhang X. (2016). Pterostilbene Inhibits Hepatocellular Carcinoma through P53/SOD2/ROS-Mediated Mitochondrial Apoptosis. Oncol. Rep..

[61] Kong Y., Chen G., Xu Z., Yang G., Li B., Wu X., Xiao W., Xie B., Hu L., Sun X. (2016). Pterostilbene Induces Apoptosis and Cell Cycle Arrest in Diffuse Large B-Cell Lymphoma Cells. Sci. Rep..

[62] Xie B., Xu Z., Hu L., Chen G., Wei R., Yang G., Li B., Chang G., Sun X., Wu H. (2016). Pterostilbene Inhibits Human Multiple Myeloma Cells via ERK1/2 and JNK Pathway In Vitro and In Vivo. Int. J. Mol. Sci..

[63] Zipper L.M., Mulcahy R.T. (2003). Erk Activation Is Required for Nrf2 Nuclear Localization during Pyrrolidine Dithiocarbamate Induction of Glutamate Cysteine Ligase Modulatory Gene Expression in HepG2 Cells. Toxicol. Sci..

[64] Lamanuzzi A., Saltarella I., Desantis V., Frassanito M.A., Leone P., Racanelli V., Nico B., Ribatti D., Ditonno P., Prete M. (2018). Inhibition of MTOR Complex 2 Restrains Tumor Angiogenesis in Multiple Myeloma. Oncotarget.

[65] Chang H.-P., Lu C.-C., Chiang J.-H., Tsai F.-J., Juan Y.-N., Tsao J.-W., Chiu H.-Y., Yang J.-S. (2018). Pterostilbene Modulates the Suppression of Multidrug Resistance Protein 1 and Triggers Autophagic and Apoptotic Mechanisms in Cisplatin-Resistant Human Oral Cancer CAR Cells via AKT Signaling. Int. J. Oncol..

[66] Mori S., Kishi S., Honoki K., Fujiwara-Tani R., Moriguchi T., Sasaki T., Fujii K., Tsukamoto S., Fujii H., Kido A. (2020). Anti-Stem Cell Property of Pterostilbene in Gastrointestinal Cancer Cells. Int. J. Mol. Sci..

[67] Wang D., Guo H., Yang H., Wang D., Gao P., Wei W. (2019). Pterostilbene, An Active Constituent of Blueberries, Suppresses Proliferation Potential of Human Cholangiocarcinoma via Enhancing the Autophagic Flux. Front. Pharmacol..

[68] Huynh T.-T., Lin C.-M., Lee W.-H., Wu A.T.H., Lin Y.-K., Lin Y.-F., Yeh C.-T., Wang L.-S. (2015). Pterostilbene Suppressed Irradiation-Resistant Glioma Stem Cells by Modulating GRP78/MiR-205 Axis. J. Nutr. Biochem..

[69] Ferrer P., Asensi M., Priego S., Benlloch M., Mena S., Ortega A., Obrador E., Esteve J.M., Estrela J.M. (2007). Nitric Oxide Mediates Natural Polyphenol-Induced Bcl-2 down-Regulation and Activation of Cell Death in Metastatic B16 Melanoma. J. Biol. Chem..

